# Supplementary figures and images for: Postharvest Properties of Ultra-Late Maturing Peach Cultivars and Their Attributions to Melting Flesh (M) Locus: Re-evaluation of M Locus in Association With Flesh Texture
Source: Front Plant Sci. 2020 Nov 26;11:554158. doi: 10.3389/fpls.2020.554158 (PMC7725752; doi:10.3389/fpls.2020.554158)

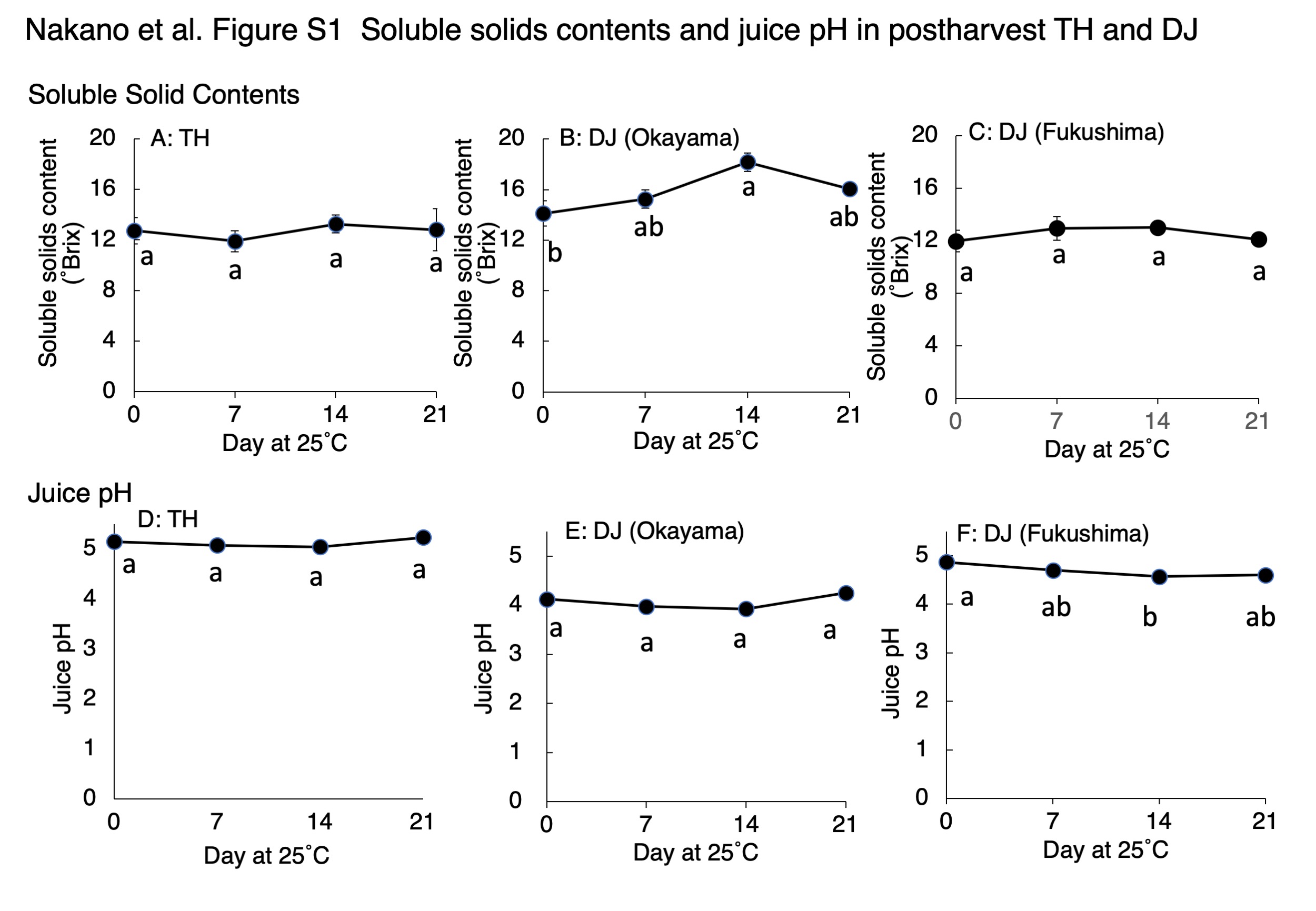

Supplement: Supplementary Figure 1 — Soluble solids contents and juice pH in postharvest TH and DJ. Postharvest changes in (A–C) soluble solids content and (D–F) juice pH in TH and DJ fruit grown in Okayama Prefecture and DJ grown in Fukushima Prefecture Japan. In (A,D), TH were harvested on November 7 from a commercial orchard in Okayama Prefecture, Japan and held at 25°C for 21 days. In (B,E), DJ from Okayama were harvested on October 12 from the Research Farm of Okayama University, Japan and held at 25°C for 21 days. In (C,F) DJ from Fukushima were harvested on October 22 from a commercial orchard in Fukushima Prefecture, Japan, followed by 2-day transport at ambient temperature to Okayama University, where fruit were held at 25°C for 21 days. Fruit were harvested at commercial maturity. Each point in (A,B,D,E) and in (C,F) represents the mean value of three and four fruits, respectively. Vertical bars indicate ± SE (n = 3–4). Statistical analysis was conducted by Tukey’s test after one-way ANOVA. Different letters indicate significant differences among measurement days by Tukey’s multiple comparison test (p < 0.05). [file Image_1.JPEG]

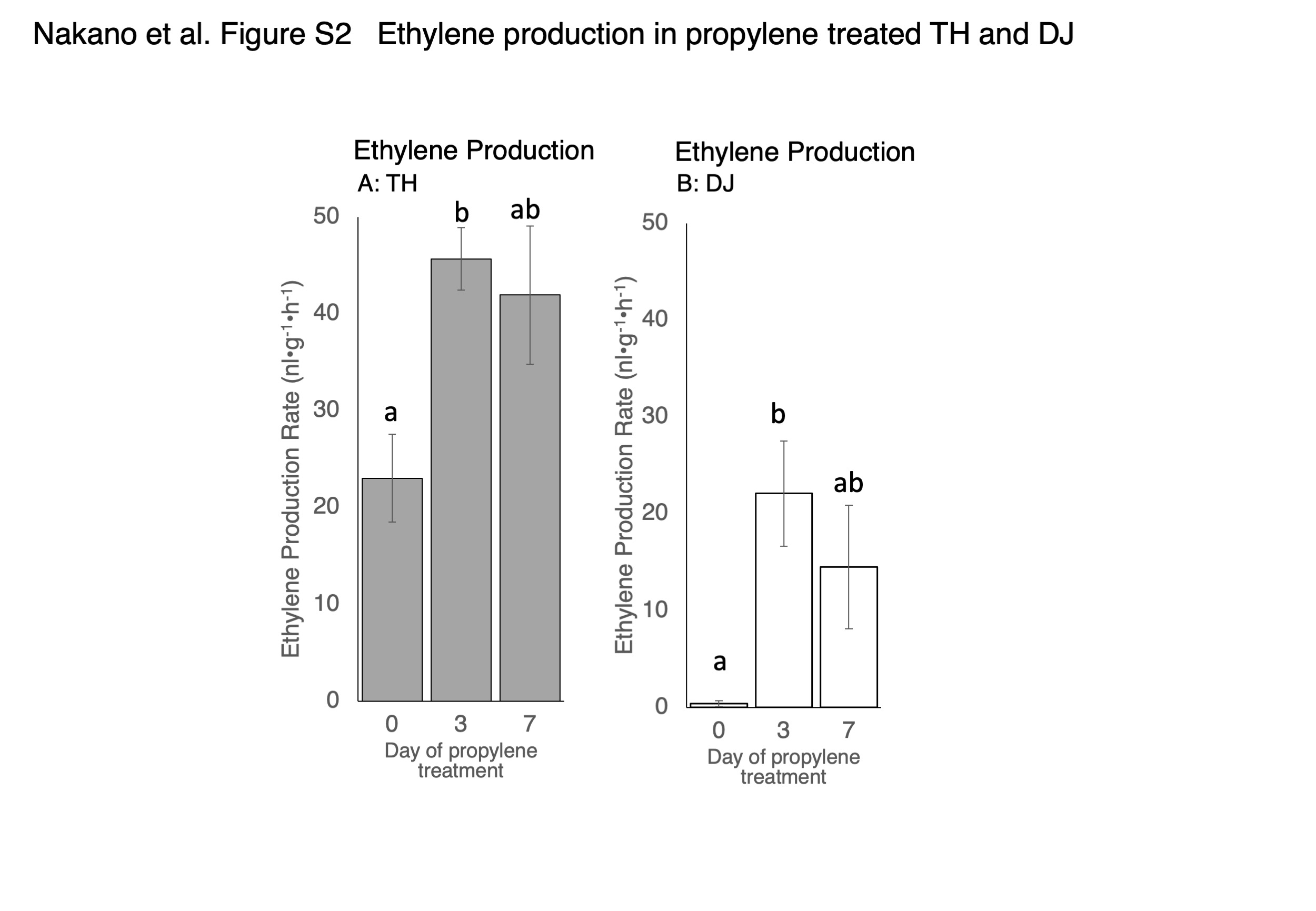

Supplement: Supplementary Figure 2 — Ethylene production in propylene treated TH and DJ. Effect of propylene treatment on postharvest ethylene production in (A) TH and (B) DJ fruit. Harvested fruit were treated with 5,000 ppm of propylene continuously for 7 days. Ethylene production was measured on days 0, 3, and 7. For (B) DJ, fruit harvested on October 12 from the Research Farm of Okayama University were used. Each point on days 0, 3, and 7 represents the mean value of four and three fruits, respectively. Vertical bars indicate ± SE (n = 3–4). Statistical analysis was conducted by Tukey’s multiple comparison test after one-way ANOVA. Different letters indicate significant differences among measurement days by Tukey’s test (p < 0.05). [file Image_2.JPEG]

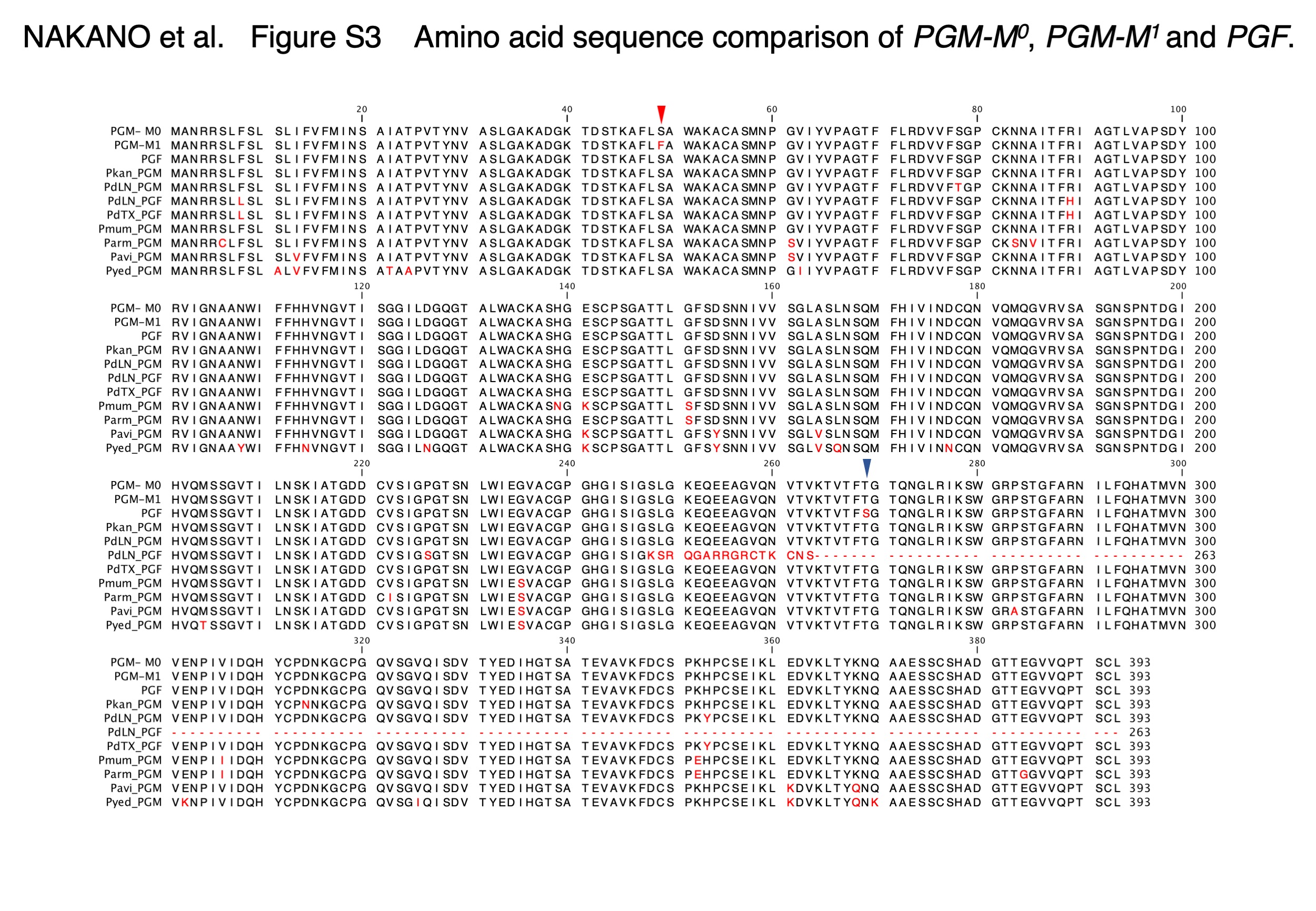

Supplement: Supplementary Figure 3 — Amino acid sequence comparison of PGM-M0, PGM-M1, and PGF. Amino acid sequences of PGM-M0, PGM-M1, and PGF of peach and PGM/F of almond “Texas” (PdTX), “Lauranne” (PdLN), P. kansuensis (Pkan), apricot (Parm), Japanese apricot (Pmum), sweet cherry (Pavi), and P. x yedoensis (Pyed) were aligned by CLC Genomics Workbench. Red and blue arrowheads denote amino acid substitution in PGM-M1 and PGF, respectively. The C-terminal region of PdLN_PGF was truncated because of the frameshift at third exon. Only one PGM/F was found in reference genome of almond “Texas,” but it could be orthologous to PGF of reference genome of “Lauranne.” PGFs of “Texas” and “Lauranne” shared some amino acid substitutions that were not conserved in PGM of “Lauranne.” [file Image_3.JPEG]

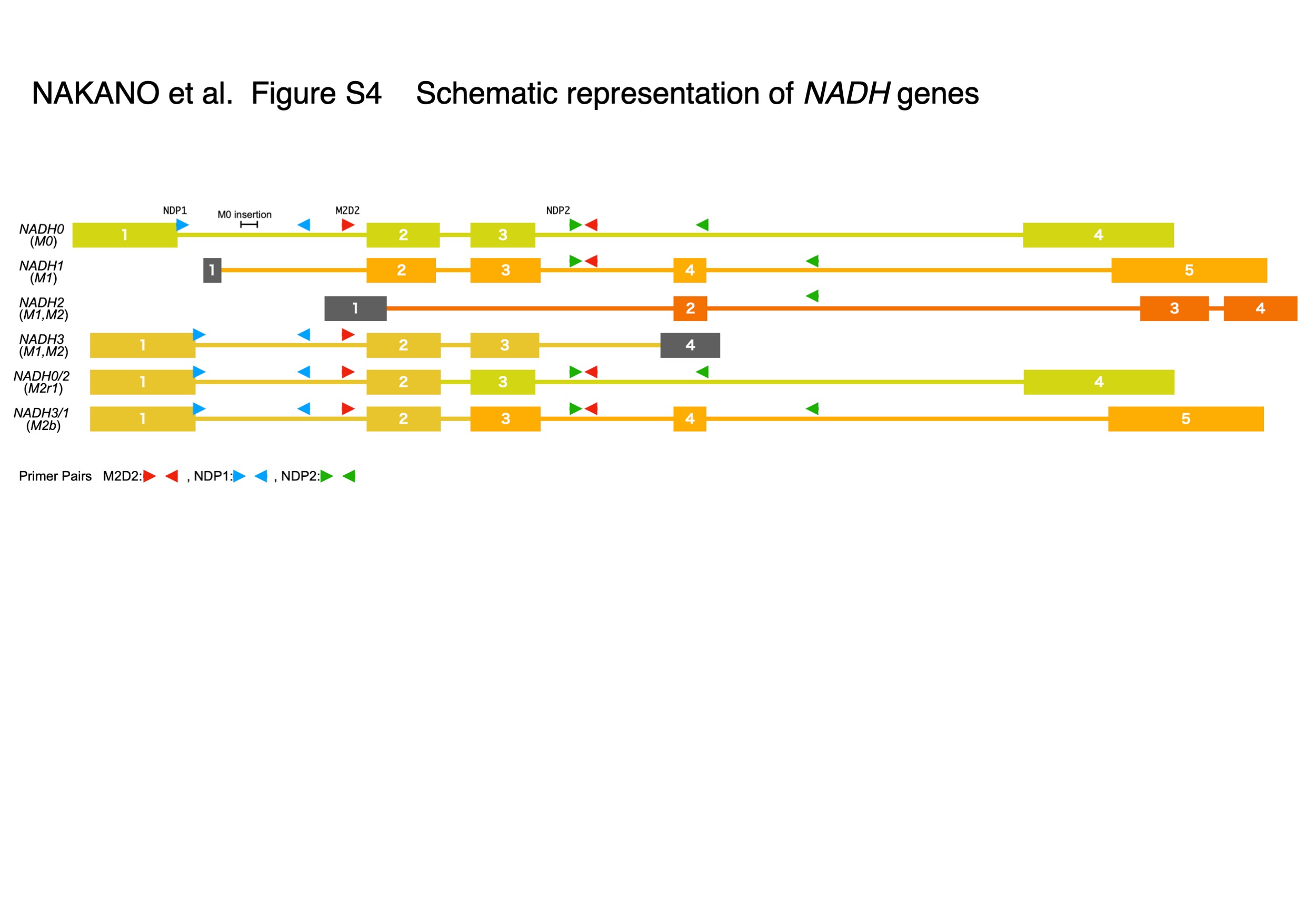

Supplement: Supplementary Figure 4 — Schematic representation of NADH genes. We compared the gene structures of NADH0–3, NADH0/2 of M2r1, and NADH3/1 of M2b. Boxes are exons. Gray exons are gene-specific sequences. Other region sequences are homologous to other genes. Sequence comparison with Arabidopsis NADH indicated that the original NADH could be composed of five exons as shown in NADH3/1 of M2b and NADH of Japanese apricot (Supplementary Figures S5, S6). [file Image_4.JPEG]

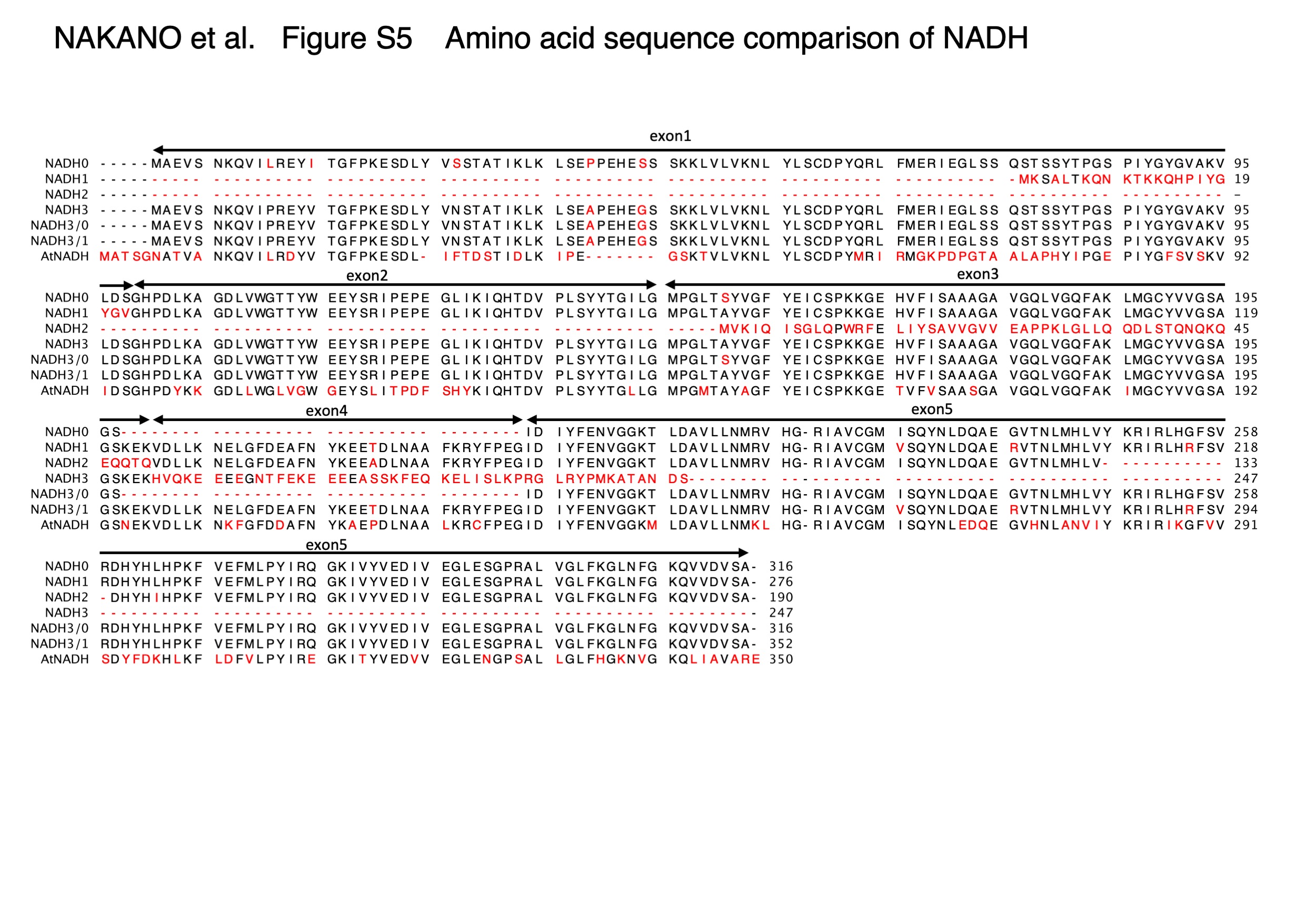

Supplement: Supplementary Figure 5 — Amino acid sequence comparison of NADH. Putative amino acid sequences of peach NADHs and Arabidopsis NADH (AtNADH; AT3G03080) were aligned by CLC Genomics Workbench. [file Image_5.JPEG]

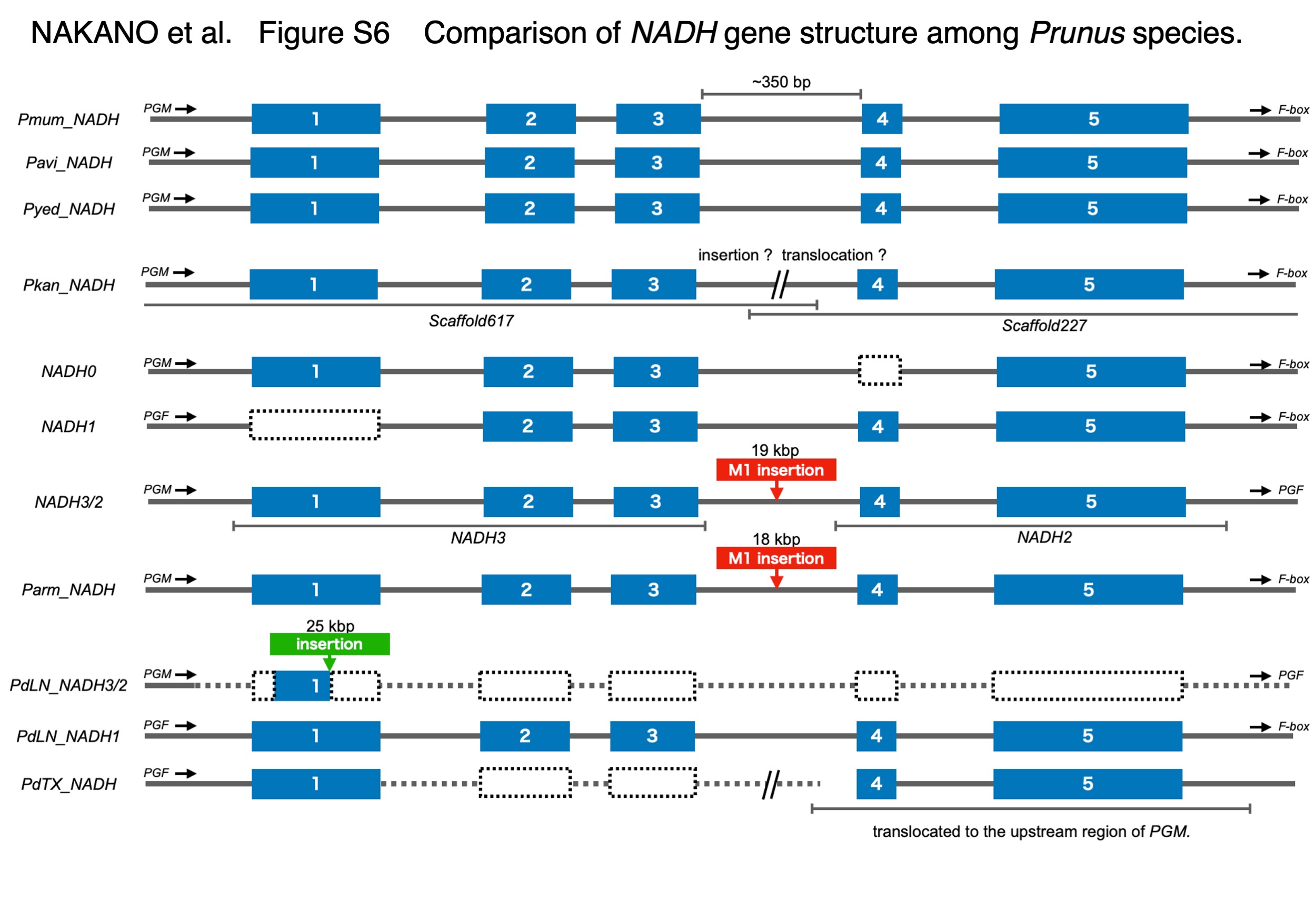

Supplement: Supplementary Figure 6 — Comparison of NADH gene structures among Prunus species. Gene structures of 11 NADHs from eight Prunus species were compared. Considering exon composition, NADH3 and NADH2 of peach were regarded as one gene (NADH3/2), although they were annotated as different genes in reference genome. Pkan NADH gene was divided into different contigs whose linkages were unknown. Disrupted structures were found in five NADHs: Pkan_NADH, NADH3/2, Parm_NADH, PdLN_NADH3/2, and PdTX_NADH. The others were likely intact structures. The M1 insertion was found at third intron of NADH3/2 and Parm_NADH. [file Image_6.JPEG]

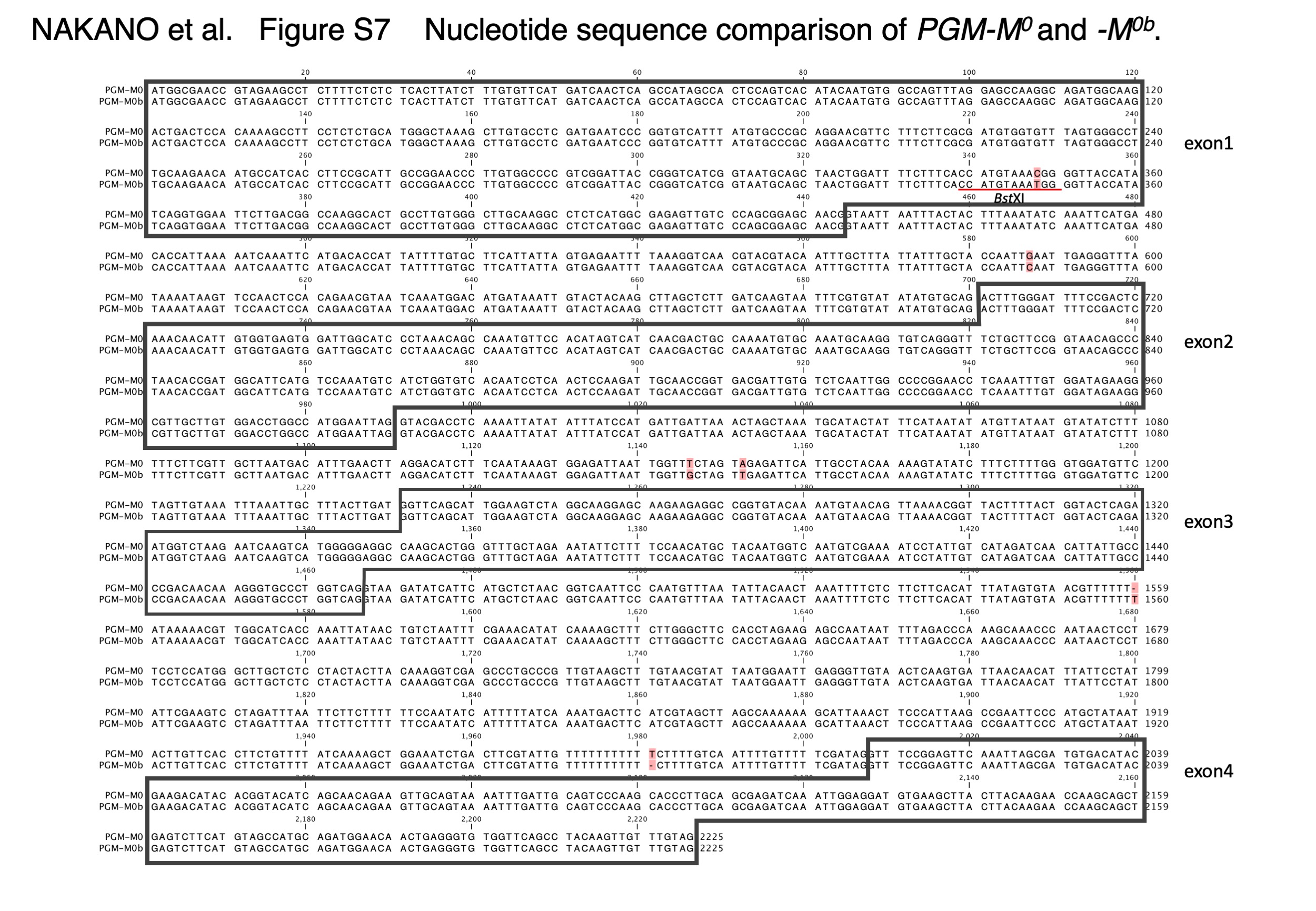

Supplement: Supplementary Figure 7 — Nucleotide sequence comparison of PGM-M0 and -M0b. Sequence comparison showed six mutations in PGM of TH (designated as PGM-M0b in this figure). Furthermore, a large insertion was predicted at the upstream region of PGM-M0b. Of the six mutations, only one was located in CDS region and it was a synonymous substitution. AAC and AAT at 346–348 encode Asn. Therefore, we did not discriminate PGM-M0 and PGM-M0b in this study. In the study of Morgutti et al. (2017), PGM-M0b was detected by CAPS analysis using BstXI. This restriction enzyme site was caused by the nucleotide substitution at 348 bp of M0b haplotype and the fragment from PGM-M0 was expected to be insensitive to BstXI. PGM-M0b could confer the MF phenotype because flesh texture was melting in both TH (M0bM3) and BT (M0bM3). Morgutti et al. (2017) proposed four alleles, PG_M, PGm, PGSH, and PGBT, at M locus from OA (M2M2), “Bolero” (M1M1), “Yumyeong” (M0M0), “Ghiaccio” (M0M0), and BT (M0bM03). Based on Figure 7, our classification suggested that PG_M and PGm were derived from M1 or M2 haplotype and these were designated as PGM-M1 and PGF, respectively, in this study (Supplementary Table S7). PGSH and PGBT could correspond to PGM-M0 and PGM-M0b, respectively. These might be strictly different alleles because of the nucleotide substitution, but flesh texture was melting in both SM (M0M3) and TH (M0bM3), suggesting that their effects on flesh texture were the same and they were not different functionally. [file Image_7.JPEG]

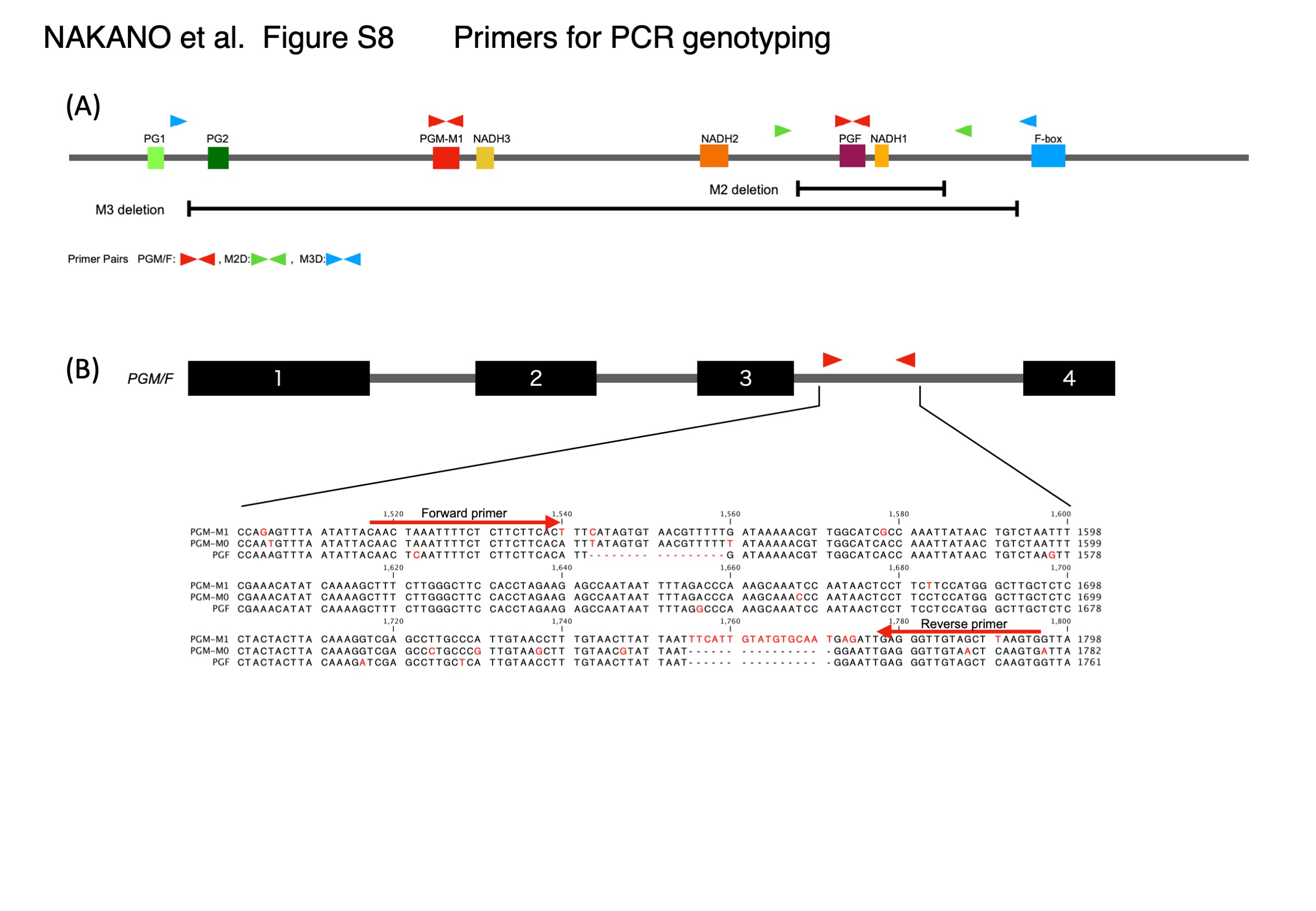

Supplement: Supplementary Figure 8 — Primers for PCR genotyping. Three primer sets were designed for the discrimination of four main haplotypes, M0, M1, M2, and M3. (A) Primer position at M1 haplotype. (B) Position of PGM/F primer set. [file Image_8.JPEG]

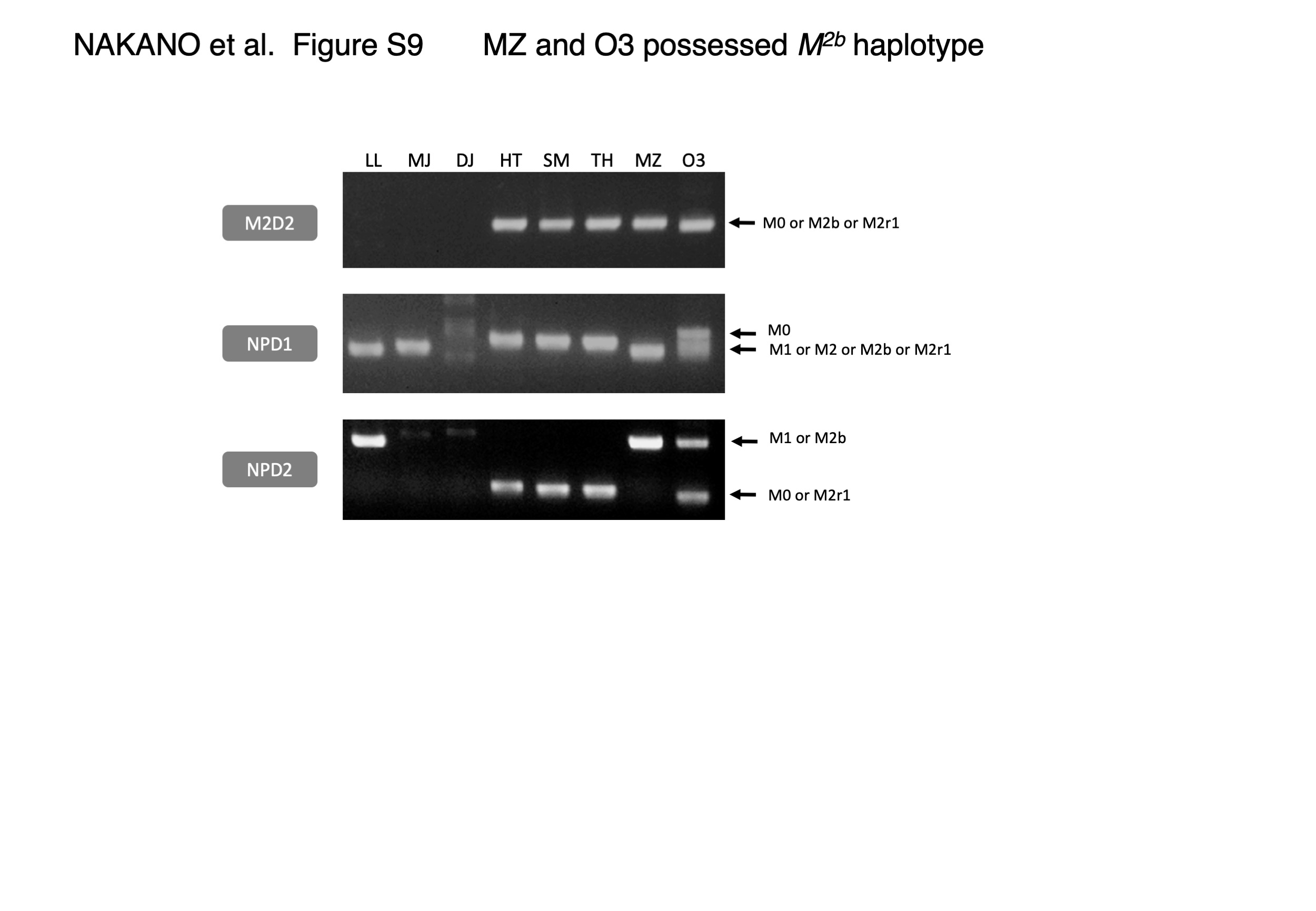

Supplement: Supplementary Figure 9 — MZ and O3 possessed M2b haplotype. MZ and O3 possessed PGM-M1 and no PGF. This pattern indicated the M2 haplotype, but no amplification to detect the M2 deletion was observed by PCR (Figure 8). In addition to M2 haplotype, we found the same combination in M2b and M2r1 haplotypes, which were rare haplotypes compared with M2. To determine the genotypes of MZ and O3, we designed three primer sets to discriminate M2, M2b, and M2r1 haplotypes on the basis of the sequences of NADH genes (Supplementary Figure S4). M2D2 amplified one fragment from M0, M2b or M2r1 haplotype. NDP1 was expected to amplify two fragments: upper for M0, and lower for M1, M2, M2b or M2r1. NDP2 was also expected to amplify two fragments: upper for M1 or M2b, and lower for M0 or M2r1. The amplification of M2D2 fragment indicated MZ had M0, M2b or M2r1 but not M1 and M2. The lower NDP1 fragment in MZ excluded the possibility of M0. Furthermore, the upper NPD2 fragment was amplified in PCR, indicating that MZ possessed M2b haplotype. O3 also had M2b, but two fragments were amplified in NDP1 and NDP2 because O3 had M0 haplotype (Figure 8). [file Image_9.JPEG]

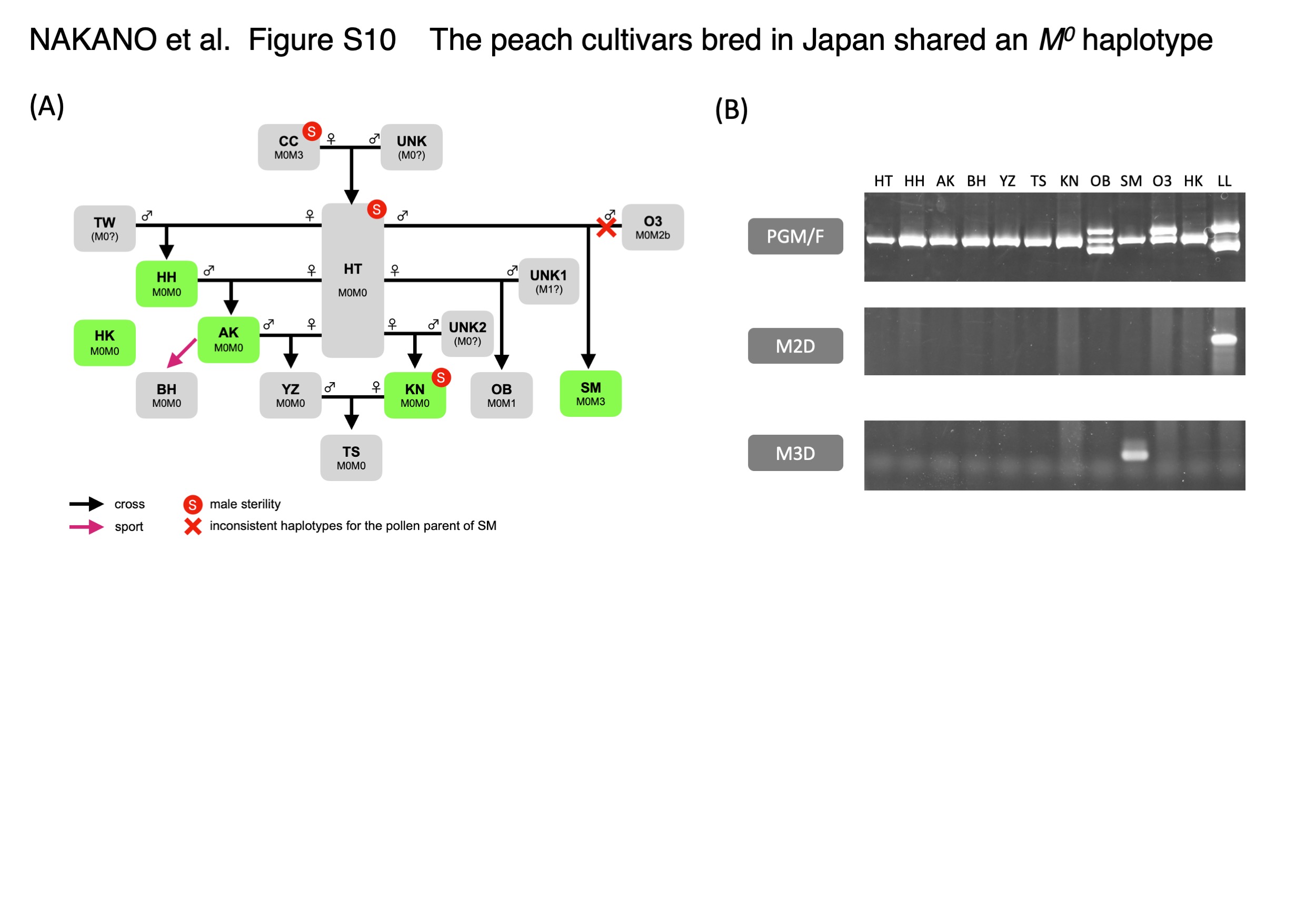

Supplement: Supplementary Figure 10 — Peach cultivars bred in Japan shared M0 haplotype. HT, Hakuto; HH, Hakuho; AK, Akatsuki; BH, Benihakuto; YZ, Yuzora; TS, Tosui; KN, KawanakajimaHakuto; OB, Okubo; SM, ShimizuHakuto; O3, Okayama-3; HK, HikawaHakuho; LL, Lovell; CC, Chinese Cling (Shanhai Suimitsuto); TW, Tachibanawase; UNK, unknown cultivar. (A) Genealogy of peach cultivars in Japan. Green cultivars were the five leading cultivars in Japan in 2016 (e-stat Japan, https://www.e-stat.go.jp/). Their cultivation areas accounted for more than 60% of the total peach cultivation area in Japan. Haplotypes in parentheses were presumed from haplotypes of offspring and another parent. (B) PCR haplotyping. CC was imported from China to Japan in the late nineteenth century. HT was reported to be found and selected as a chance seedling of CC in 1899 (Yamamoto et al., 2003a). HT was frequently used as seed parent in breeding programs and many Japanese peach cultivars were the progeny of HT, as described Supplementary Figure S10A. We selected 11 Japanese MF cultivars and carried out PCR genotyping to determine the genotypes of their M loci. All cultivars shared M0 haplotype and all cultivars except OB and SM were M0 homozygous. OB was M0M1 and SM was M0M3. SM was found as a chance seedling at a mixed orchard of HT and O3. SSR analysis supported the hypothesis that SM was a progeny of HT (Yamamoto et al., 2003a). Because of male sterility of HT, O3 had been regarded as the pollen parent of SM. M0 haplotype of SM was inherited from HT and seed parent should possess M3 haplotype, because HT was M0 homozygous. These indicated that O3 was not the parent of SM. [file Image_10.JPEG]

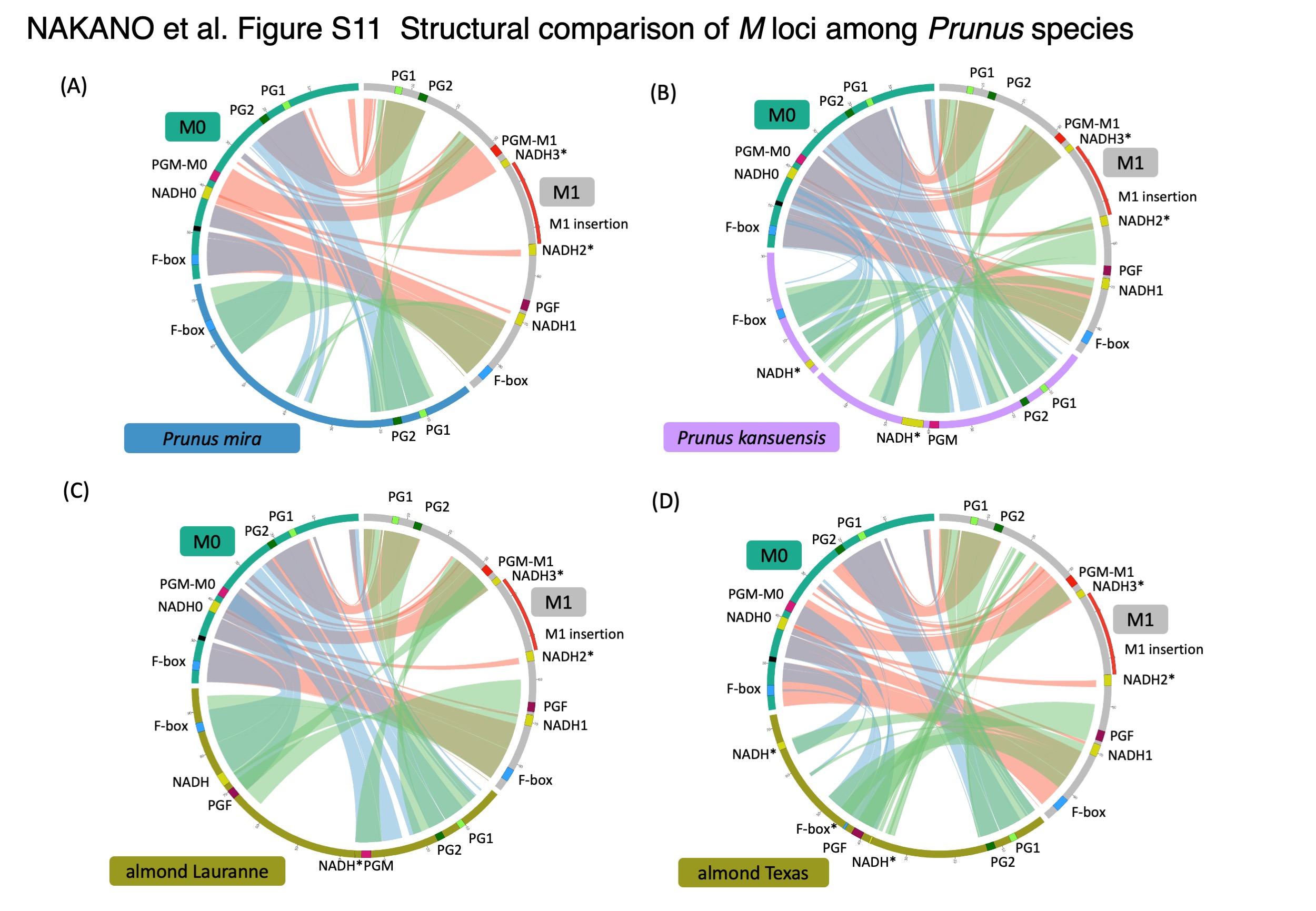

Supplement: Supplementary Figure 11 — Structural comparison of M loci among Prunus species. Circos plots show sequence similarities among M0, M1, and M haplotypes of other Prunus species. Ribbons link homologous regions between haplotypes. ∗Indicates genes that did not have intact CDS sequence. PG1 of P. mume was translocated to the > 2 Mbp downstream region. To determine the M locus region, reference genomes of almond (“Lauranne” and “Texas”), P. kansuensis, P. mira, Japanese apricot, apricot, sweet cherry, and P. x yedoensis were searched by Blastn analysis using PG1, PG2, PGM/F, NADH, and F-box genes as query. M locus was found at the right arm of chromosome 4 or LG3 in all Prunus species except P. kansuensis, in which no pseudomolecule was released. Nucleotide sequences of M locus were compared by nucmer and the relationships were drawn by Circos. [file Image_11.JPEG]

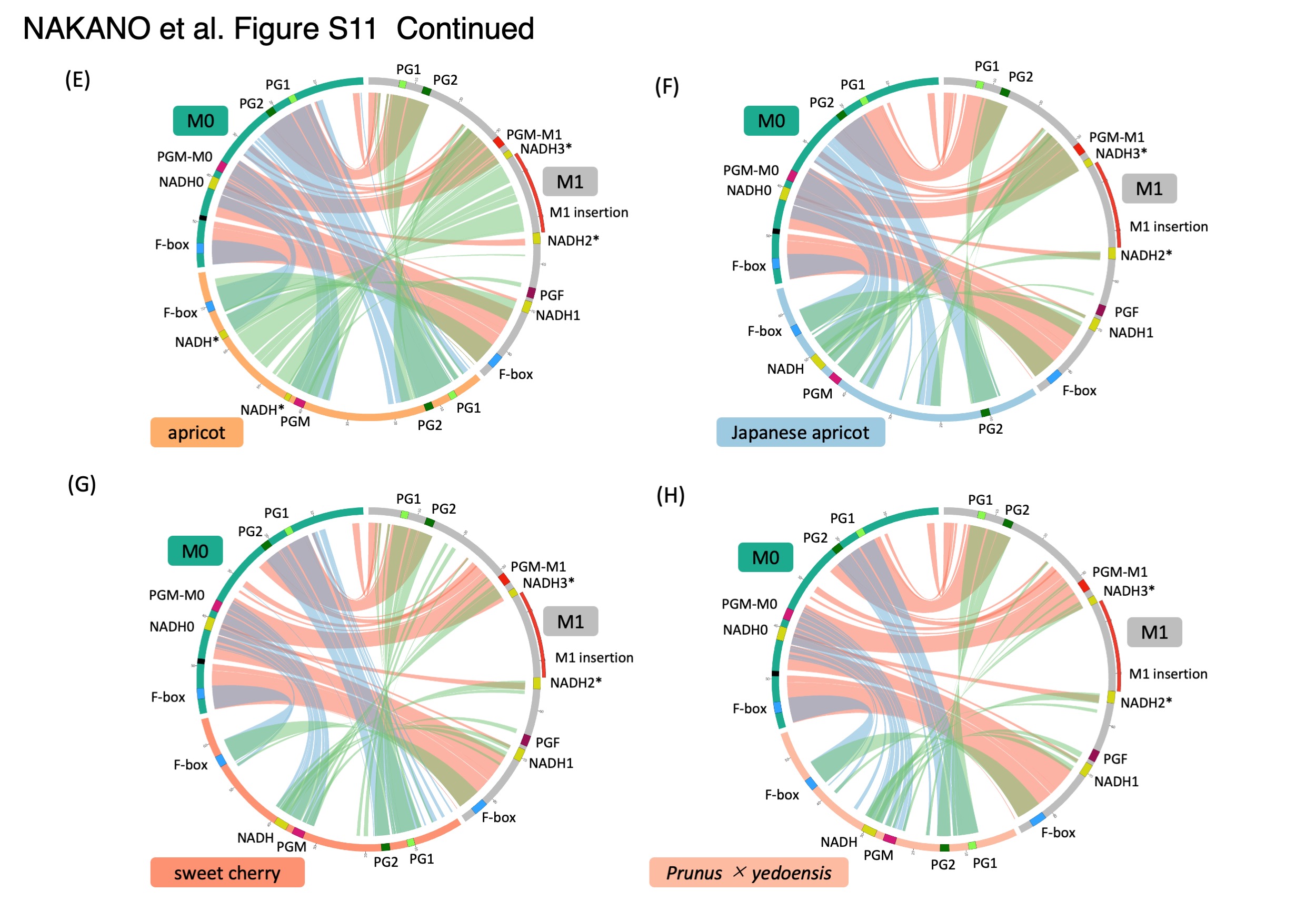

Supplement: Supplementary file 12 [file Image_12.JPEG]
