# Supplementary material for: Postharvest Properties of Ultra-Late Maturing Peach Cultivars and Their Attributions to Melting Flesh (M) Locus: Re-evaluation of M Locus in Association With Flesh Texture
Source: Front Plant Sci. 2020 Nov 26;11:554158. doi: 10.3389/fpls.2020.554158 (PMC7725752; doi:10.3389/fpls.2020.554158)
Supplement: Supplementary Table 2 — Relationships between flesh penetration force measured by the system used in this study and other fruit maturity indexes. [file Data_Sheet_1.DOCX]

Supplementary Material

**Legends for Supplemental Tables and Figures**

Table S1

Climate conditions in peach production areas in Okayama and Fukushima Prefectures, Japan in 2018

Table S2

Relationships between flesh penetration force measured by the system used in this study and other fruit maturity indexes.

Table S3

Primers used in this study

Table S4

Comparison of SNP number in regions from *PG1* to *PG2*

Intergenic region 1 was from end of *PG1* to 19,026,185 bp (outside H3 deletion region).

Intergenic region 2 was from 19,026,186 bp (inside H3 deletion region) to start of *PG2.*

Values in parentheses are those of heterozygous SNPs.

Table S5

*M* genotypes of 412 peach accessions

Table S6

Differences in flesh texture predicted by genotype and reported phenotype

* The reported phenotype matched the predicted one in this study.

The 11 accessions showed differences between predicted and reported phenotypes. All except 'Early Gold' (EG) were reported by Yoon et al. (2006) and Cao et al. (2016).

EG was a canning peach and its parent was 'Nishiki' (NK). EG should possess at least one *M^2^* haplotype because NK was an *M^2^* homozygote, but resequencing analysis showed that EG was *M^1^M^1^*.

Table S7

Correlation of *PGM/F* genes in this study with those in previous reports

*PG^BT^* in Morgutti et al. (2017) was *Bst*XI-sensitive (Fig. S6).

Table S8

Correlation of haplotypes in this study with those in previous reports

**M^0^* was misidentified as H_2_ haplotype in Gu et al. (2016)

f haplotype structure in Morgutti et al. (2017) was postulated from *M^1^* haplotype. *PG* gene composition was corrected but the genome structure was not the same as *M^0^* haplotype in this study.

*PGM-M^0^* and *PGM-M^1^* were allelic.

Figure S1

Soluble solids contents and juice pH in postharvest TH and DJ

Postharvest changes in (A, B, C) soluble solids content and (D, E, F) juice pH in TH and DJ fruit grown in Okayama Prefecture and DJ grown in Fukushima Prefecture Japan. In (A) and (D), TH were harvested on November 7 from a commercial orchard in Okayama Prefecture, Japan and held at 25˚C for 21 days. In (B) and (E), DJ from Okayama were harvested on October 12 from the Research Farm of Okayama University, Japan and held at 25˚C for 21 days. In (C) and (F), DJ from Fukushima were harvested on October 22 from a commercial orchard in Fukushima Prefecture, Japan, followed by two-day transport at ambient temperature to Okayama University, where fruit were held at 25˚C for 21 days. Fruit were harvested at commercial maturity. Each point in (A, B, D, E) and in (C. F) represents the mean value of three and four fruits, respectively. Vertical bars indicate ± SE (n = 3 to 4). Statistical analysis was conducted by Tukey’s test after one-way ANOVA. Different letters indicate significant differences among measurement days by Tukey’s multiple comparison test (*p* < 0.05).

Figure S2

Ethylene production in propylene treated TH and DJ

Effect of propylene treatment on postharvest ethylene production in (A) TH and (B) DJ fruit. Harvested fruit were treated with 5,000 ppm of propylene continuously for seven days. Ethylene production was measured on days 0, 3, and 7. For (B) DJ, fruit harvested on October 12 from the Research Farm of the Faculty of Agriculture, Okayama University were used. Each point on day 0 and day 3, 7 represents the mean value of four and three fruits, respectively. Vertical bars indicate ± SE (n = 3 to 4). Statistical analysis was conducted by Tukey’s multiple comparison test after one-way ANOVA. Different letters indicate significant differences among measurement days by Tukey’s test (*p* < 0.05).

Figure S3

Amino acid sequence comparison of PGM-*M^0^*, PGM-*M^1^*, and PGF.

Amino acid sequences of PGM-*M^0^*, PGM-*M^1^*, and PGF of peach and PGM/F of almond ‘Texas’ (PdTX), ‘Lauranne’ (PdLN), *P. kansuensis* (Pkan), apricot (Parm), Japanese apricot (Pmum), sweet cherry (Pavi), and *P.* x *yedoensis* (Pyed) were aligned by CLC Genomics Workbench. Red and blue arrowheads denote amino acid substitution in PGM-*M^1^* and PGF, respectively.

The C-terminal region of PdLN_PGF was truncated because of the frameshift at third exon. Only one PGM/F was found in reference genome of almond ‘Texas’, but it could be orthologous to PGF of reference genome of ‘Lauranne’. PGFs of ‘Texas’ and ‘Lauranne’ shared some amino acid substitutions that were not conserved in PGM of ‘Lauranne’.

Figure S4

Schematic representation of *NADH* genes.

We compared the gene structures of *NADH0–3*, *NADH0/2* of *M^2r1^*, and *NADH3/1* of *M^2b^*. Boxes are exons. Grey exons are gene-specific sequences. Other region sequences are homologous to other genes.

Sequence comparison with Arabidopsis *NADH* indicated that the original *NADH* could be composed of five exons as shown in *NADH3/1* of *M^2b^* and *NADH* of Japanese apricot (Figs. S5 and S6).

Figure S5

Amino acid sequence comparison of NADH.

Putative amino acid sequences of peach NADHs and Arabidopsis NADH (AtNADH; AT3G03080) were aligned by CLC Genomics Workbench.

Figure S6

Comparison of *NADH* gene structures among *Prunus* species.

Gene structures of 11 *NADH*s from eight *Prunus* species were compared. Considering exon composition, *NADH3* and *NADH2* of peach were regarded as one gene (*NADH3*/*2*), although they were annotated as different genes in reference genome. *Pkan NADH* gene was divided into different contigs whose linkages were unknown.

Disrupted structures were found in five *NADH*s: *Pkan_NADH*, *NADH3/2*, *Parm_NADH*, *PdLN_NADH3/2*, and *PdTX_NADH*. The others were likely intact structures. The M1 insertion was found at third intron of *NADH3/2* and *Parm_NADH*.

Figure S7

Nucleotide sequence comparison of *PGM-M^0^* and -*M^0b^*.

Sequence comparison showed six mutations in *PGM* of TH (designated as *PGM-M^0b^* in this figure). Furthermore, a large insertion was predicted at the upstream region of *PGM-M^0b^*. Of the six mutations, only one was located in CDS region and it was a synonymous substitution. AAC and AAT at 346–348 encode Asn. Therefore, we did not discriminate *PGM-M^0^* and *PGM-M^0b^* in this study. In the study of Morgutti et al. (2017), *PGM-M^0b^* was detected by CAPS analysis using BstXI. This restriction enzyme site was caused by the nucleotide substitution at 348 bp of *M^0b^* haplotype and the fragment from *PGM-M^0^* was expected to be insensitive to *Bst*XI. *PGM-M^0b^* could confer the MF phenotype because flesh texture was melting in both TH (*M^0b^M^3^*) and BT (*M^0b^M^3^*).

Morgutti et al. (2017) proposed four alleles, *PG_M*, *PG^m^*, *PG^SH^*, and *PG^BT^*, at *M* locus from OA (*M^2^M^2^*), Bolero (*M^1^M^1^*), Yumyeong (*M^0^M^0^*), Ghiaccio (*M^0^M^0^*), and BT (*M^0b^M^0^*). Based on Fig. 7, our classification suggested that *PG_M* and *PG^m^* were derived from *M^1^* or *M^2^* haplotype and these were designated as *PGM-M^1^* and *PGF*, respectively, in this study (Table S7). *PG^SH^* and *PG^BT^* could correspond to *PGM-M^0^* and *PGM-M^0b^*, respectively. These might be strictly different alleles because of the nucleotide substitution, but flesh texture was melting in both SM (*M^0^M^3^*) and TH (*M^0b^M^3^*), suggesting that their effects on flesh texture were the same and they were not different functionally.

Figure S8

Primers for PCR genotyping.

Three primer sets were designed for the discrimination of four main haplotypes, *M^0^*, *M^1^*, *M^2^*, and *M^3^*. (A) Primer position at *M^1^* haplotype. (B) Position of PGM/F primer set.

Figure S9

MZ and O3 possessed *M^2b^* haplotype.

MZ and O3 possessed *PGM*-*M^1^* and no *PGF*. This pattern indicated the *M^2^* haplotype, but no amplification to detect the *M^2^* deletion was observed by PCR (Fig. 8). In addition to *M^2^* haplotype, we found the same combination in *M^2b^* and *M^2r1^* haplotypes, which were rare haplotypes compared with *M^2^*. To determine the genotypes of MZ and O3, we designed three primer sets to discriminate *M^2^*, *M^2b^*, and *M^2r1^* haplotypes on the basis of the sequences of *NADH* genes (Fig. S4). M2D2 amplified one fragment from *M^0^*, *M^2b^* or *M^2r1^* haplotype. NDP1 was expected to amplify two fragments: upper for *M^0^*, and lower for *M^1^*, *M^2^*, *M^2b^* or *M^2r1^*. NDP2 was also expected to amplify two fragments: upper for *M^1^* or *M^2b^*, and lower for *M^0^* or *M^2r1^*. The amplification of M2D2 fragment indicated MZ had *M^0^,* *M^2b^* or *M^2r1^* but not *M^1^* and *M^2^*. The lower NDP1 fragment in MZ excluded the possibility of *M^0^*. Furthermore, the upper NPD2 fragment was amplified in PCR, indicating that MZ possessed *M^2b^* haplotype. O3 also had *M^2b^*, but two fragments were amplified in NDP1 and NDP2 because O3 had *M^0^* haplotype (Fig. 8).

Figure S10

Peach cultivars bred in Japan shared *M^0^* haplotype.

HT: ‘Hakuto’, HH: ‘Hakuho’, AK: ‘Akatsuki’, BH: ‘Benihakuto’, YZ: ‘Yuzora’, TS: ‘Tosui’, KN: ‘KawanakajimaHakuto’, OB: ‘Okubo’, SM: ‘ShimizuHakuto’, O3: ‘Okayama-3’, HK, ‘HikawaHakuho’, LL: ‘Lovell’, CC: ‘Chinese Cling (Shanhai Suimitsuto)’, TW: ‘Tachibanawase’, UNK: unknown cultivar

(A) Genealogy of peach cultivars in Japan. Green cultivars were the five leading cultivars in Japan in 2016 (e-stat Japan, <https://www.e-stat.go.jp/>). Their cultivation areas accounted for more than 60% of the total peach cultivation area in Japan. Haplotypes in parentheses were presumed from haplotypes of offspring and another parent.

(B) PCR haplotyping. CC was imported from China to Japan in the late 19th century. HT was reported to be found and selected as a chance seedling of CC in 1899 (Yamamoto et al., 2003). HT was frequently used as seed parent in breeding programs and many Japanese peach cultivars were the progeny of HT, as described Figure S10A. We selected 11 Japanese MF cultivars and carried out PCR genotyping to determine the genotypes of their *M* loci. All cultivars shared *M^0^* haplotype and all cultivars except OB and SM were *M^0^* homozygous. OB was *M^0^M^1^* and SM was *M^0^M^3^*. SM was found as a chance seedling at a mixed orchard of HT and O3. SSR analysis supported the hypothesis that SM was a progeny of HT. Because of male sterility of HT, O3 had been regarded as the pollen parent of SM. *M^0^* haplotype of SM was inherited from HT and seed parent should possess *M^3^* haplotype, because HT was *M^0^* homozygous. These indicated that O3 was not the parent of SM.

Yamamoto, T., K. Mochida and T. Hayashi (2003) Shanhai Suimitsuto, One of the Origins of Japanese Peach Cultivars. J. Japan. Soc. Hort. Sci. 72: 116-121.

Figure S11

Structural comparison of *M* loci among *Prunus* species

Circos plots show sequence similarities among *M^0^*, *M^1^*, and *M* haplotypes of other *Prunus* species. Ribbons link homologous regions between haplotypes. * indicates genes that did not have intact CDS sequence. *PG1* of *P. mume* was translocated to the > 2M bp downstream region.

To determine the *M* locus region, reference genomes of almond (‘Lauranne’ and ‘Texas’), *P. kansuensis*, *P. mira*, Japanese apricot, apricot, sweet cherry, and *P.* x *yedoensis* were searched by Blastn analysis using *PG1*, *PG2*, *PGM/F*, *NADH*, and *F-box* genes as query. *M* locus was found at the right arm of chromosome 4 or LG3 in all *Prunus* species except *P.* *kansuensis*, in which no pseudomolecule was released. Nucleotide sequences of *M* locus were compared by nucmer and the relationships were drawn by Circos.
